# Radiomics for differentiating radiation-induced brain injury from recurrence in gliomas: systematic review, meta-analysis, and methodological quality evaluation using METRICS and RQS

**DOI:** 10.1007/s00330-025-11401-x

**Published:** 2025-02-12

**Authors:** Burak Kocak, Ismail Mese, Ece Ates Kus

**Affiliations:** 1 Department of Radiology, University of Health Sciences, Basaksehir Cam and Sakura City Hospital, Istanbul, Turkey; 2 Department of Radiology, Uskudar State Hospital, Istanbul, Turkey; 3 Institute of Neuroradiology, Klinikum Lippe, Lemgo, Germany

**Keywords:** Radiomics, Artificial intelligence, Machine learning, Deep learning, Glioma

## Abstract

**Objective:**

To systematically evaluate glioma radiomics literature on differentiating between radiation-induced brain injury and tumor recurrence.

**Methods:**

Literature was searched on PubMed and Web of Science (end date: May 7, 2024). Quality of eligible papers was assessed using METhodological RadiomICs Score (METRICS) and Radiomics Quality Score (RQS). Reliability of quality scoring tools were analyzed. Meta-analysis, meta-regression, and subgroup analysis were performed.

**Results:**

Twenty-seven papers were included in the qualitative assessment. Mean average METRICS score and RQS percentage score across three readers was 57% (SD, 14%) and 16% (SD, 12%), respectively. Score-wise inter-rater agreement for METRICS ranged from poor to excellent, while RQS demonstrated moderate to excellent agreement. Item-wise agreement was moderate for both tools. Meta-analysis of 11 eligible studies yielded an estimated area under the receiver operating characteristic curve of 0.832 (95% CI, 0.757–0.908), with significant heterogeneity (*I*^2^ = 91%) and no statistical publication bias (*p* = 0.051). Meta-regression did not identify potential sources of heterogeneity. Subgroup analysis revealed high heterogeneity across all subgroups, with the lowest *I*^2^ at 68% in studies with proper validation and higher quality scores. Statistical publication bias was generally not significant, except in the subgroup with the lowest heterogeneity (*p* = 0.044). However, most studies in both qualitative analysis (26/27; 96%) and primary meta-analysis (10/11; 91%) reported positive effects of radiomics, indicating high non-statistical publication bias.

**Conclusion:**

While a good performance was noted for radiomics, results should be interpreted cautiously due to heterogeneity, publication bias, and quality issues thoroughly examined in this study.

**Key Points:**

***Question*** *Radiomic literature on distinguishing radiation-induced brain injury from glioma recurrence lacks systematic reviews and meta-analyses that assess methodological quality using radiomics-specific tools*.

***Findings*** *While the results are encouraging, there was substantial heterogeneity, publication bias toward positive findings, and notable concerns regarding methodological quality*.

***Clinical relevance*** *Meta-analysis results need cautious interpretation due to significant problems detected during the analysis (e.g., suboptimal quality, heterogeneity, bias), which may help explain why radiomics has not yet been translated into clinical practice*.

**Graphical Abstract:**

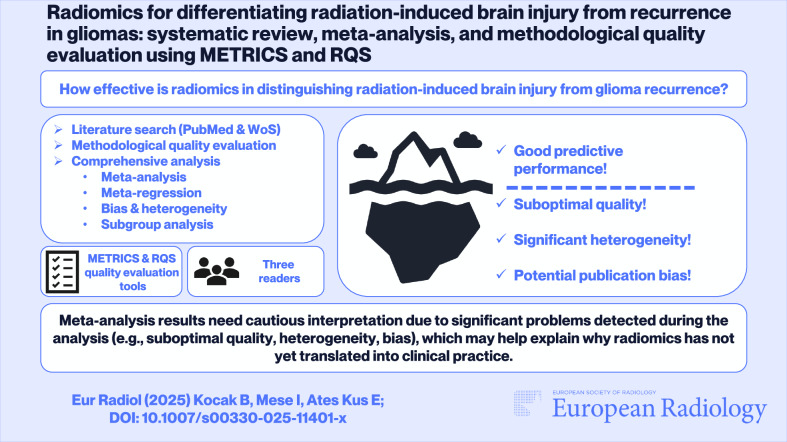

## Introduction

Gliomas encompass a broad spectrum of central nervous system tumors, classified by the World Health Organization (WHO) into grades 1 to 4 [1, 2]. While high-grade gliomas (grades 3 and 4) are typically malignant and associated with high recurrence and mortality rates, low-grade gliomas (grades 1 and 2) are generally less aggressive, though they still carry the risk of recurrence. Standard postoperative treatment for high-grade gliomas includes radiotherapy and temozolomide chemotherapy [3, 4], which may also be used for low-grade gliomas separately or in combination [5, 6]. However, this treatment strategy often leads to radiation-induced brain injury, such as pseudoprogression and radiation necrosis [7]. Treatment-related effects in the form of radiation-induced brain injury may indicate the treatment’s efficacy, necessitating only symptomatic management rather than regimen adjustments [8]. Conversely, tumor recurrence typically requires more aggressive anti-cancer treatment and potential re-operation [9]. Thus, the timely and accurate detection of tumor recurrence is crucial for optimizing clinical treatment plans.

Differentiating between radiation-induced brain injury and actual tumor recurrence on diagnostic imaging is challenging [10]. MRI is the recommended standard diagnostic method for evaluating tumor response post-treatment, as per the Response Assessment in Neuro Oncol guidelines [11, 12]. However, both conditions typically appear as enhanced lesions in MRI, complicating the differentiation [13]. Advanced imaging techniques, particularly perfusion MRI, have shown promise in addressing this challenge [14]. Among the perfusion MRI techniques, dynamic susceptibility contrast (DSC) MR perfusion has demonstrated superior diagnostic performance in differentiating glioma recurrence from radiation-induced changes [15]. Despite the promising results, variability in imaging parameters and threshold values necessitates further investigation and standardization [15]. FDG-PET and FET-PET have also demonstrated potential in distinguishing these conditions [16, 17]. Despite this promise, consensus on their efficacy remains elusive due to limited studies and variability in scanning protocols [18].

In this context, radiomics may offer diagnostic solutions by extracting a large number of features from medical images and converting them into high-dimensional data [19–21]. This approach is assumed to capture the spatial heterogeneity of tumors from conventional medical images and reveals hidden information that non-radiomics methods might miss. When combined with machine learning algorithms, radiomics can provide valuable prognostic or predictive insights, thus aiding clinical decision-making in tumor diagnosis, staging, and prognosis [19]. However, concerns have been raised about the methodological quality of radiomics research due to the complexity of the technique and lack of standardization in many aspects [22], which has primarily been assessed using the Radiomics Quality Score (RQS) [23]. Recently, new tools such as the METhodological RadiomICs Score (METRICS) have emerged [24], offering a more nuanced assessment of methodological rigor by focusing on different aspects of study quality [25].

The main objective of this study was to systematically evaluate the methodological quality of peer-reviewed literature on glioma radiomics, particularly focusing on studies differentiating between radiation-induced brain injury and tumor recurrence. Given the lack of literature using quality assessment tools specific to radiomics (e.g., METRICS and RQS), we primarily aimed to address this gap. Additionally, we conducted a meta-analysis to synthesize the findings from the eligible studies. A secondary objective was to provide insights into the score-wise and item-wise reliability of the quality scoring tools used, namely METRICS and RQS.

## Methods

PRISMA checklists are provided in the Online Supplement (Supplementary Checklist S1 and S2).

### Eligibility

To be eligible for this work, papers were required to be original research articles on radiomics research, including handcrafted and deep learning-based approaches. Papers differentiating radiation-induced brain injury from recurrence in gliomas were included. Studies assessing the preoperative prediction performance of these conditions were excluded. In addition, publications without full text, without clear descriptions of tumors, written in a language other than English, and with a retraction notice were excluded from this work.

### Literature search

**Fig. 1:**
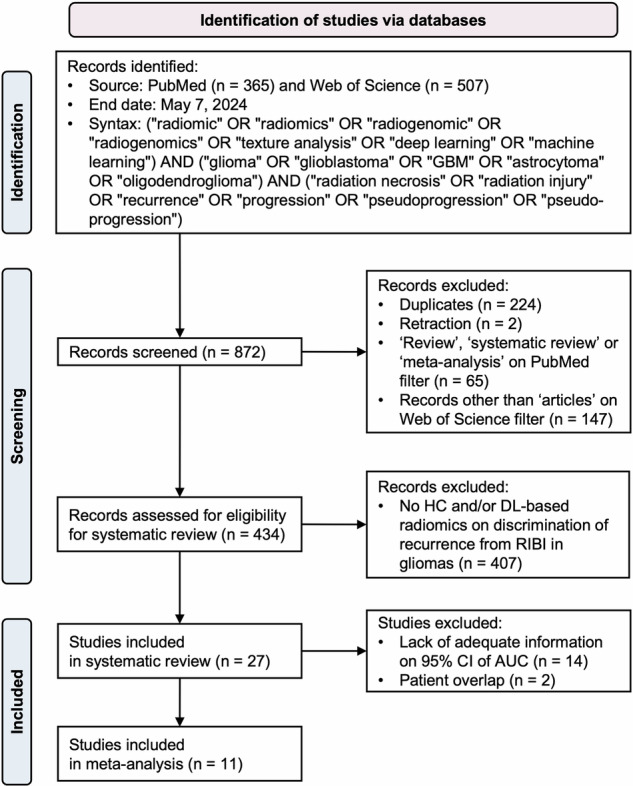
Modified PRISMA flow diagram. HC, handcrafted; DL, deep learning; RIBI, radiation-induced brain injury; CI, confidence interval; AUC, area under the receiver operating characteristic curve

Literature was searched on PubMed and Web of Science (end date: May 7, 2024). The search syntax is presented in Fig. 1.

Two readers independently performed the literature search and screening. Titles, abstracts, and full texts (only if necessary) were screened based on the eligibility criteria defined. Full texts of the eligible papers were subsequently reviewed by three independent readers for qualitative and quantitative (i.e., meta-analysis) assessment.

### Qualitative assessment

Methodological quality of the eligible papers was assessed using METRICS [24] and RQS [23]. The choice of these two tools was based on the lack of other radiomics-specific quality scoring alternatives (to the best of our knowledge) and their complementary nature, as suggested by a recent hypothetical analysis [25]. Three readers independently performed the assessments. All of the readers were radiologists: Reader 1 with at least 6; Reader 2 and 3 with at least 2 years of experience in radiomics. Before the evaluation, all readers were exposed to these two tools’ original papers [23, 24]. For training, the readers discussed items of both tools in a question-and-answer style, without referring to the eligible papers. Average scores and collective or cumulative evaluations were presented where appropriate.

Proposed by Lambin et al in 2017, the RQS is composed of 16 items and has been widely used for the assessment of the quality of radiomics research [23]. In the original paper and the literature, total RQS points (range, −8 to +36) are non-linearly scaled to 100% by which negative total scores correspond to 0%, which was considered throughout this paper for presenting the results.

Recently published in 2024, METRICS is a 30-item quality scoring tool [24]. METRICS items and their weights were determined with a modified Delphi approach by a broad international panel. It has nine categories and five conditions to cover all aspects of radiomics research including handcrafted and deep learning-based approaches. The final METRICS score is calculated on a percentage scale (0 to 100%) based on the linear scaling method and conditions selected. As recommended, the METRICS-based assessment was performed on its website which allows the calculation of final score based on selected conditions and consensus-based item weights.

In addition, the following baseline study characteristics were extracted by consensus of three readers: author names, publication year, journal name, journal category, number of centers, number of patients, imaging modality used in radiomics analysis, details on sequence or tracer, radiomic approach, performance of the best model, validation type, WHO grade of tumor, patient age range, treatment strategy, and comparison with a reference method. Furthermore, the overall study conclusions regarding radiomics or its effects were evaluated to provide a basis for assessing the presence of non-statistical publication bias (i.e., the tendency to publish positive results).

### Quantitative assessment

From the eligible studies, the ones reporting the upper and lower bounds of the 95% confidence interval (CI) of the area under the receiver operating characteristic curve (AUC) were subsequently included in the meta-analysis. These bounds were directly used in meta-analysis or used to estimate the standard error for meta-regression. Inferences were based on the restricted maximum likelihood method and identity link function. Heterogeneity between studies was evaluated with *I*^2^ index (cut-off = 50%). Additionally, meta-regression and subgroup analysis were performed to evaluate potential factors related to between-study heterogeneity and their effect on the final performance estimate. In case of high heterogeneity, regardless of the meta-regression analysis results, we further explored variables by conducting subgroup analyses to decrease the heterogeneity considering the variables regarding the validation methods (i.e., studies with internal or external hold-out set), methodological quality as assessed by dedicated tools (i.e., high-quality studies based on METRICS and RQS; above the mean score), and WHO grading (i.e., grade 3 or 4 gliomas). To assess statistical publication bias, funnel plot asymmetry was tested using Egger’s method. In case of high heterogeneity, the multiplicative overdispersion method was used. Due to the low agreement among statistical methods, non-statistical publication bias was also evaluated as recommended [26], as described above in the qualitative assessment part. Summary estimate and prediction interval of the AUC were calculated. Eligible papers in the meta-analysis were evaluated for overlapping patient data by checking dataset origin institutions. If unavailable, the first author’s institution was used. The dataset generation timeframe was also considered. Meta-analysis was conducted in JASP (version 0.19.1 [Apple Silicon]) based on the R packages ‘metamisc,’ ‘metafor,’ and ‘stats.’

### Statistical analysis

Statistical analysis was conducted in JASP (version 0.19.1 [Apple Silicon]). Normality of the distribution was assessed using Shapiro–Wilk test. Differences were analyzed with parametric or non-parametric tests based on the distribution. Scores were compared using Friedman test, with post hoc analysis for pairwise comparisons and Bonferroni correction. Correlation was analyzed using Kendall’s tau. Reliability of scores was analyzed using intraclass correlation coefficient (ICC), according to the Shrout and Fleiss convention [27] and relevant guidelines [28]. In line with our data and results presented throughout the study, two different types of ICC were calculated as the interpretation of ICC values largely depends on the use case and context. Reliability analysis of categorical evaluations was performed using Fleiss’ kappa. Statistical significance threshold was 0.05.

## Results

### Literature search

Syntax-based search resulted in 872 records from the two databases. Screening with eligibility criteria revealed 27 eligible papers with 2402 patients for systematic review and qualitative assessment [29–55]. Of those, 13 papers were eligible for the quantitative evaluation (i.e., meta-analysis), but two had potential patient overlaps. As a result, 11 papers were subsequently included in the final quantitative assessment.

The literature search is summarized in Fig. 1.

### Baseline characteristics

About two-thirds of the papers (18/27; 67%) were published between 2020 and 2023. Journal types were as follows: clinical (10/27; 37%), imaging (7/27; 26%), multi-disciplinary (4/27; 15%), health informatics (3/27; 11%), neuroscience (1/27; 4%), and others (2/27; 7%).

Mean number of patients in the papers was 89 (standard deviation, 43). The age range was clearly within the adult population in 22 studies (81%). However, two studies included pediatric patients (age < 18), while the age range was not clearly defined in the remaining three studies.

Sixteen studies (59%) included only WHO grade 4 gliomas, while seven studies (26%) included both WHO grade 3 and 4 gliomas, categorized as high-grade gliomas. In contrast, the remaining four studies (15%) also included low-grade gliomas (WHO grades 1 and/or 2) besides high-grade gliomas.

MRI only (23/27; 85%) was the most frequently used imaging modality in the radiomics analysis, followed by PET only (3/27; 11%) and PET + MRI (1/27; 4%). Ten studies used traditional MRI only (37%) in their radiomic analysis.

The handcrafted-only approach (16/27; 59%) dominated the radiomic approaches, and it was followed by deep learning-only (8/27; 30%) and combined handcrafted-deep learning approaches (3/27; 11%). In studies using the handcrafted approach, the most common feature classes in the final models were gray level co-occurrence matrix (45% of all features), first-order (19%), and gray level run length matrix (16%), in decreasing order.

Only 37% (10/27) of the studies were based on multiple institutions (i.e., multi-center). Only more than half of the papers had separate test data: internal hold-out (9/27; 33%) and external (6/27; 22%). About one-third analyzed their data with resampling strategies only: cross-validation (9/27; 33%) and bootstrapping (1/27; 4%). Two studies (7%) presented their results using their whole data.

Except for one study, all the remaining papers (26/27; 96%) concluded that radiomics is promising, without any overall negative result, indicating non-statistical publication bias. However, about half of the papers (13/27; 48%) did reach their conclusion without any reference method for comparison (e.g., radiologists’ evaluation).

Baseline study characteristics are presented in Table 1 and Online Supplement (Supplementary Table S1).

**Table 1:** Baseline characteristics of 27 papers eligible for qualitative evaluation

Authors	No. of patients	No. of centers	Imaging modality	Radiomic approach	Validation method	Performance of best model^a^	Conclusion for radiomics
Ahrari et al [54]	85	2	PET	HC	External	AUC = 0.834	Positive
Elshafeey et al [50]	98	3	MRI	HC	CV	AUC = 100%^b^	Positive
Gao and Wang et al [38]	56	2	MRI	HC	Hold-out	AUC = 94%	Positive (w/o comp)
Hagiwara et al [44]	169	> 1	MRI	HC	Hold-out	AUC = 0.64	Positive
Kim et al [45]	95	2	MRI	HC	External	AUC = 0.85	Positive
Lohmann et al [42]	34	1	PET	HC	Hold-out	AUC = 0.74	Positive
Park et al [37]	127	2	MRI	HC	External	AUC = 0.80	Positive
Sun et al [35]	77	1	MRI	HC	Hold-out	ACC = 72.8%	Positive
Wang et al [46]	160	1	PET + MRI	HC	Hold-out	AUC = 0.914	Positive (w/o comp)
Zhang et al [36]	51	1	MRI	HC + DL	Bootstrapping	AUC = 0.999	Positive (w/o comp)
Zhang et al [40]	126	1	MRI	HC	Hold-out	AUC = 0.94	Positive (w/o comp)
Chen et al [39]	22	1	MRI	HC	Whole data	AUC = 0.892	Positive (w/o comp)
Akbari et al [43]	83	2	MRI	HC + DL	External	AUC = 0.80	Positive (w/o comp)
Bacchi et al [33]	55	1	MRI	DL	Hold-out	AUC = 0.80	Positive (w/o comp)
Gao and Xiao et al [34]	146	1	MRI	DL	Hold-out	AUC = 0.958; 0.915 (ib)	Positive
Jang et al [51]	78	2	MRI	DL	External	AUC = 0.83	Positive
Kebir et al [55]	14	1	PET	HC	Whole data	NPV = 75%	Positive
Lee et al [41]	43	1	MRI	DL	CV	AUC = 0.81	Positive (w/o comp)
Li et al [32]	84	1	MRI	DL	CV	AUC = 0.947	Positive (w/o comp)
Moassefi et al [29]	124	1	MRI	DL	CV	AUC = 88.72%	Positive (w/o comp)
Patel et al [49]	76	1	MRI	HC	CV	AUC = 0.80	Positive
Siakallis et al [47]	83	1	MRI	HC	CV	ACC = 94.7%	Positive
Zhang et al [52]	79	1	MRI	DL	CV	AUC = 0.87	Positive (w/o comp)
Leone et al [30]	105	1	MRI	HC + DL	CV	AUC = 0.59 (DL)	Negative
McKenney et al [53]	74	> 1	MRI	HC	CV	AUC = 0.89	Positive
Bani-Sadr et al [31]	76	1	MRI	HC	Hold-out	AUC = 0.85	Positive (w/o comp)
Jang et al [48]	182	> 3	MRI	DL	CV + External	AUC (CV) = 0.86	Positive (w/o comp)

*CV* cross-validation, *NPV* negative predictive value, *ACC* accuracy, *AUC* area under the curve, *HC* handcrafted, *DL* deep learning, *ib* image-based, *w/o comp* without comparison to a reference method

^a^ Presented as seen in the source articles. Selected and prioritized according to validation techniques and their importance in the following order (from highest to lowest importance): external testing, internal testing (i.e., hold-out), resampling methods, and whole data

^b^ Cross-validation results are considered due to unclear descriptions for the external dataset

### Qualitative assessment

The mean average METRICS score of the three readers (i.e., mean of average of three readers for all publications) was 57%, with a standard deviation of 14%. On the other hand, the mean average RQS percentage score was 16%, with a standard deviation of 12%. Statistical distribution of average scores was normal for both tools. Compared to linear scaling (i.e., [points + 8]/44*100) [25], non-linearly scaled RQS underestimated the overall quality of the papers with a median and interquartile range of 14% and 3%, respectively. Further descriptive statistics are presented in Table 2.

**Table 2:** Descriptive statistics of three readers’ final scores

Scoring tools	Readers	Mean	SD	Median	IQR	Minimum	Maximum	*p*-value of Shapiro–Wilk
METRICS^a^	Reader 1	59	14	60	18	18	86	0.548
	Reader 2	62	15	65	13	24	84	0.153
	Reader 3	49	16	51	15	4	71	0.087
	Average	57	14	59	12	15	77	0.066
RQS^a^	Reader 1	16	13	11	25	0	36	0.004
	Reader 2	16	14	11	22	0	42	0.017
	Reader 3	16	13	17	22	0	36	0.011
	Average	16	12	16	19	0	36	0.094

*METRICS* methodological radiomics score, *RQS* radiomics quality score, *CI* confidence interval, *SD* standard deviation, *IQR* interquartile range

^a^ Scores are presented on percentage scale and rounded to nearest whole number

**Fig. 2:**
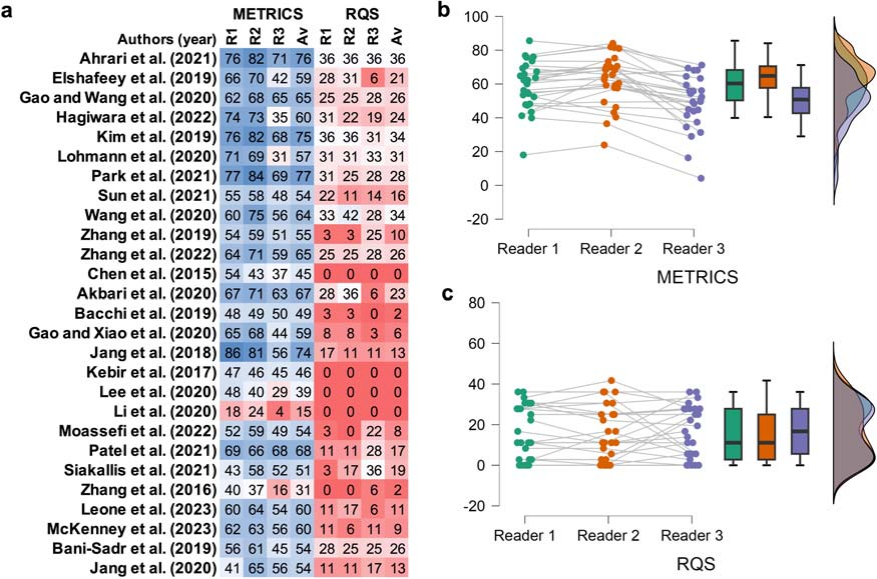
Heatmap (**a**) for evaluation scores of individual readers and their averages per publication. Raincloud plots (**b**, **c**) for distribution of individual scores of readers per publication. Scores of both quality scoring tools are on percentage scale. METRICS, methodological radiomics score; RQS, radiomics quality score; R1, reader 1; R2, reader 2; R3, reader 3; Av, average

Readers’ individual and average scores, along with their distribution, are presented in Fig. 2. Statistical distribution of scores was normal for METRICS (*p* > 0.05) and skewed for RQS (*p* < 0.05) for each reader’s evaluation. Sphericity was violated for both tools. METRICS scores among three readers were statistically significantly different (Friedman test, *p* < 0.001), while RQS scores were not (Friedman test, *p* = 0.661). Post hoc pairwise comparisons are presented in Table 3.

**Table 3:** Post hoc comparisons of individual readers’ METRICS and RQS scores

Scoring tool	Readers		p_bonf_
METRICS^a^	Reader 1	Reader 2	0.049
		Reader 3	< 0.001
	Reader 2	Reader 3	< 0.001
RQS^a^	Reader 1	Reader 2	1.000
		Reader 3	1.000
	Reader 2	Reader 3	1.000

*METRICS* methodological radiomics score, *RQS* radiomics quality score, *bonf* Bonferroni

^a^ Conover’s post hoc test was performed for the Friedman test

**Fig. 3:**
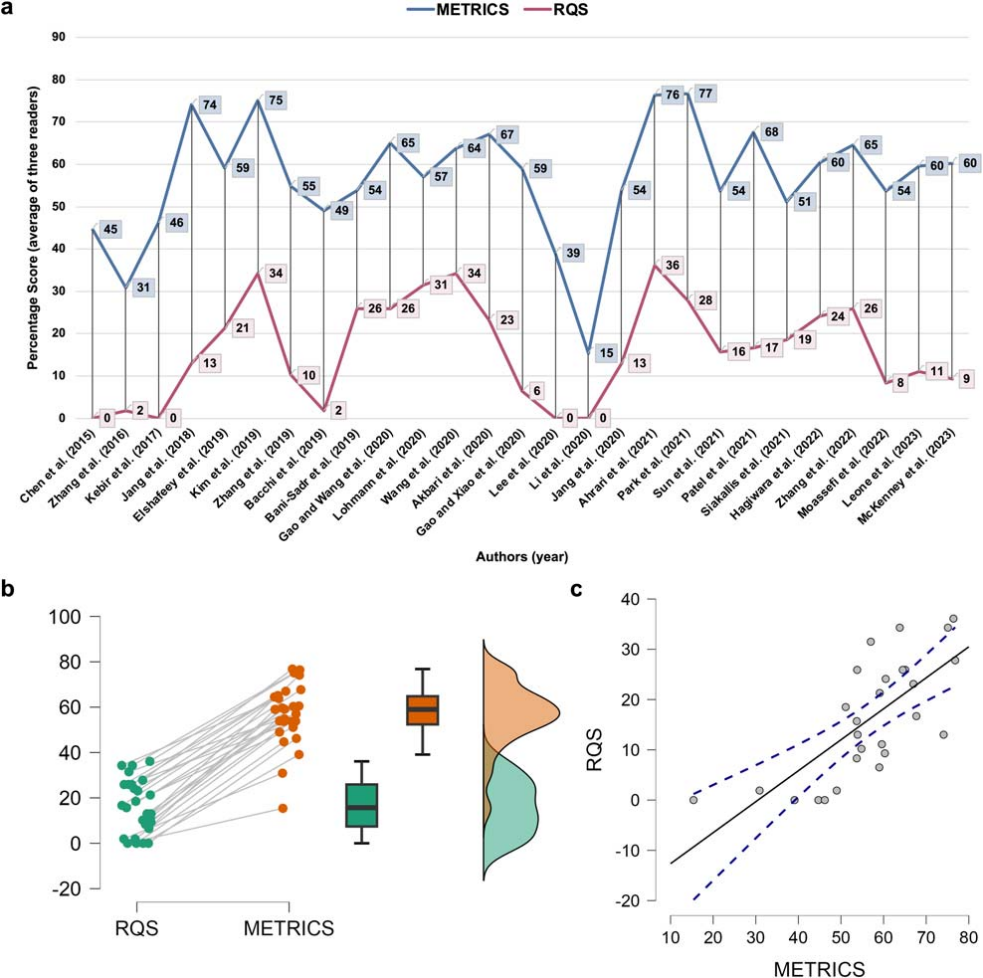
Line chart (**a**) for average scores of three readers per publication, sorted according to publication year. Raincloud plot (**b**) for distribution of average scores of three readers per publication. Scatter plot (**c**) for correlation of average scores in quality scoring tools. Scores of both quality scoring tools are on a percentage scale. METRICS, methodological radiomics score; RQS, radiomics quality score

Based on the average scores per publication, the difference between METRICS and RQS scores is illustrated in Fig. 3a. Average METRICS and RQS scores in paired analysis are statistically significantly different, *p* < 0.001 (Fig. 3b). There was no statistically significant correlation between publication year and the scores of both tools (Kendall’s tau B, 0.235 for METRICS; 0.195 for RQS; *p* > 0.05 for both). On the other hand, METRICS and RQS percentage scores were strongly correlated, with Kendall’s tau B of 0.569, *p* < 0.001 (Fig. 3c).

**Fig. 4:**
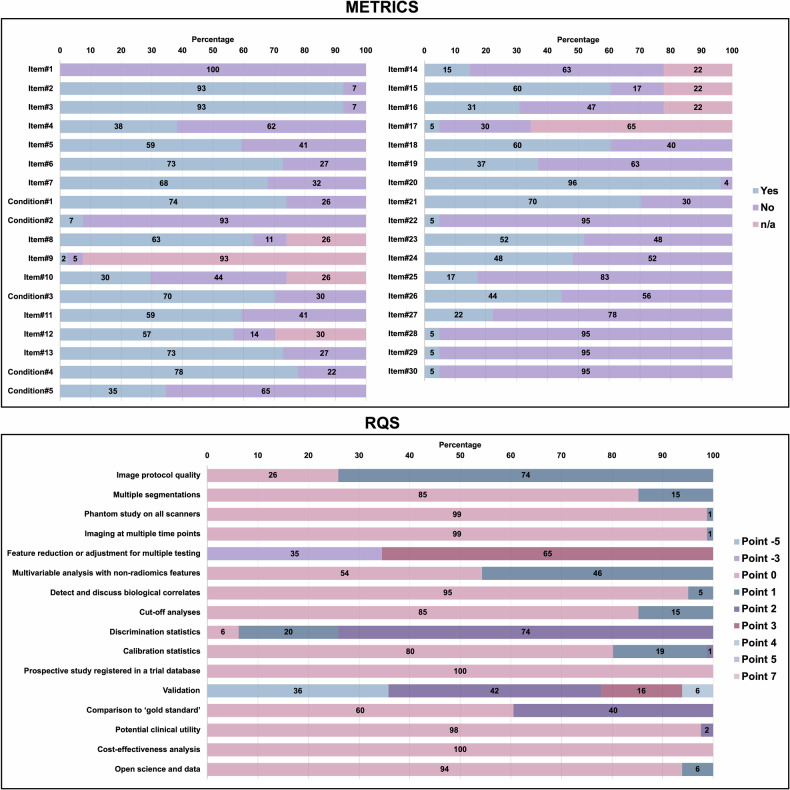
Item-wise evaluation based on three readers’ independent assessment and collective input, in percentage scale. Numeric values represent the percentage of occurrences of evaluation categories for each item. Please note that, due to automatic rounding, some totals may not precisely sum to 100% or may slightly exceed this value. METRICS, methodological radiomics score; RQS, radiomics quality score

Pooling all readers’ evaluations in the analysis, Fig. 4 shows item-wise assessment in both quality scoring tools on a percentage scale. For METRICS, there was no single positive evaluation for item#1 (i.e., adherence to radiomics and/or machine learning-specific checklists or guidelines). Also, 95% of the assessments about open science were negative: item#28 (i.e., data availability), item#29 (i.e., code availability), and item#30 (i.e., model availability). Moreover, item#22 (i.e., calibration assessment) was negative in 95% of the evaluations. In addition to these, the following items resulted in a negative evaluation in at least 50% of the assessments when excluding the non-applicable ones: item#4 (i.e., multi-center), item#9 (i.e., formal evaluation of fully automated segmentation), item#10 (i.e., test set segmentation masks produced by a single reader or automated tool), item#14 (i.e., removal of non-robust features), item#16 (i.e., appropriateness of dimensionality compared to data size), item#17 (i.e., robustness assessment of end-to-end deep learning pipelines), item#19 (i.e., handling of confounding factors), item#24 (i.e., comparison with a non-radiomic approach or proof of added clinical value), item#25 (i.e., comparison with simple or classical statistical models), item#26 (i.e., internal testing), and item#27 (i.e., external testing). For RQS, there was almost no positive evaluation for ‘phantom study on all scanners,’ ‘imaging at multiple time points,’ ‘prospective study registered in a trial database,’ and ‘cost-effectiveness analysis.’ Also, more than 90% of assessments in the following items lacked a positive evaluation: ‘detect and discuss biological correlates,’ ‘potential clinical utility,’ and ‘open science and data.’ Only the following items received at least a positive evaluation in more than 50% of assessments: ‘image protocol quality,’ ‘feature reduction or adjustment for multiple testing,’ ‘discrimination statistics,’ and ‘validation’ (in terms of having at least one separate validation set).

Reliability analysis based on the final METRICS and RQS scores of three readers is presented in Table 4. METRICS achieved poor to moderate agreement based on a single measurement, but moderate to excellent agreement based on three readers’ average measurements. On the other hand, RQS achieved moderate to good and good to excellent agreement for the same measurements, respectively.

**Table 4:** Reliability analysis based on final scores of three readers

Scoring tools	Intraclass correlation				Agreement category^b,c^
	Type^a^	Point estimate	Lower 95% CI	Upper 95% CI	
METRICS	ICC (2,1)	0.628	0.272	0.824	Poor to moderate
	ICC (2,3)	0.835	0.529	0.933	Moderate to excellent
RQS	ICC (2,1)	0.709	0.531	0.842	Moderate to good
	ICC (2,3)	0.879	0.773	0.941	Good to excellent

*METRICS* methodological radiomics score, *RQS* radiomics quality score, *CI* confidence interval, *ICC* intraclass correlation

^a^ According to Shrout and Fleiss (1979) convention

^b^ Interpretation scale was as follows: ICC < 0.50, poor; 0.50 ≤ ICC < 0.75, moderate; 0.75 ≤ ICC < 0.90, good; and ICC ≥ 0.90, excellent

^c^ Interpreted based on 95% confidence intervals as recommended

**Fig. 5:**
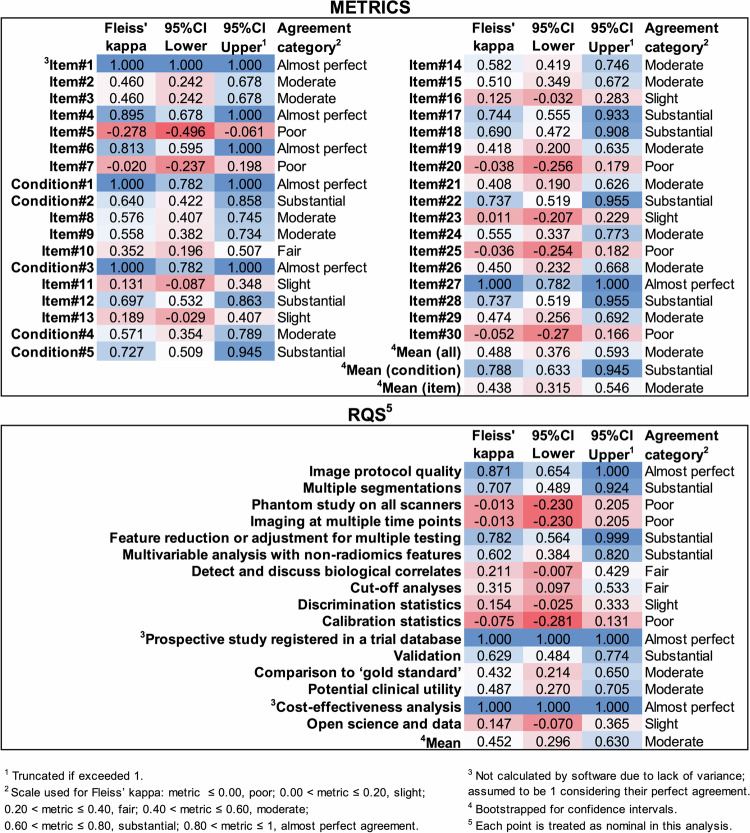
Item-wise reliability analysis based on three reader’s evaluation. METRICS, methodological radiomics score; RQS, radiomics quality score; CI, confidence interval

Item-wise reliability analysis is presented in Fig. 5. Including (i.e., conditions + items) and excluding (i.e., items only) conditions, the mean Fleiss’ kappa for METRICS was 0.488 (95% CI, 0.376–0.593) and 0.438 (95% CI, 0.315–0.546), respectively. The mean Fleiss’ kappa for the five conditions of METRICS was 0.788 (95% CI, 0.633–0.945). The mean Fleiss’ kappa for RQS was 0.452 (95% CI, 0.296–0.630). The item-wise agreement based on the Fleiss’ kappa was moderate for both METRICS and RQS.

For transparency, METRICS and RQS assessments of the three readers are presented in the Online Supplement (Supplementary Figs. S1–6).

### Quantitative assessment

For the 11 studies with 1029 patients eligible for meta-analysis, the estimated AUC was 0.832 (95% CI, 0.757–0.908). There was a significant heterogeneity between studies (*I*^2^ = 91%). No statistically significant statistical publication bias was observed according to the funnel plot asymmetry test (Egger’s test with multiplicative overdispersion, *p* = 0.051). However, most studies (10/11; 91%) reported positive effects of radiomics (i.e., non-statistical publication bias or the tendency of publishing positive results), similar to the qualitative assessment part of the study (26/27; 96%).

Extensive univariate meta-regression analysis showed no variable significantly impacting the estimate and heterogeneity (*p* > 0.05 and *I*^2^ > 50% for all variables). Meta-regression results are presented in Table 5. All subgroup analyses showed promising results similar to the primary analysis. However, none of these analyses decreased the heterogeneity indicator (i.e., *I*^2^) below 50%. The least heterogeneous study population was achieved by combining both high-quality studies and the ones with internal or external hold-out sets (*I*^2^ = 68%). However, in this subgroup analysis, there appeared to be statistically significant statistical publication bias according to the funnel plot asymmetry test (Egger’s test with multiplicative overdispersion, *p* = 0.044).

**Table 5:** Meta-regression results

Variables	Subcategories	Estimate	Standard error	95% Confidence interval		*p*-value
				Lower	Upper	
Year	-	−0.019	0.017	−0.058	0.019	0.285
Journal type	Health informatics (ref)	-	-	-	-	-
	Others	−0.081	0.16	−0.474	0.312	0.632
	Clinical	−0.184	0.125	−0.491	0.122	0.192
	Multi-disciplinary	−0.109	0.133	−0.435	0.216	0.442
	Imaging	−0.035	0.117	−0.322	0.251	0.774
Number of patients	-	−61.300	8.505 × 10^−^^4^	−0.002	0.001	0.517
Number of centers	Single-center (ref)	-	-	-	-	-
	Multi-center	0.011	0.073	−0.153	0.175	0.882
Imaging modality used in radiomics (group#1)	PET (ref)	-	-	-	-	-
	MRI	−0.003	0.132	−0.300	0.295	0.983
Imaging modality used in radiomics (group#2)	Not MRI (ref)	-	-	-	-	-
	Traditional MRI only	0.034	0.144	0.820	−0.299	0.367
	Traditional and advanced MRI	−0.024	0.138	0.865	−0.343	0.294
Radiomic approach (best)	Deep learning (ref)	-	-	-	-	-
	Handcrafted	0.072	0.074	−0.095	0.238	0.354
Validation (group#1)	No hold-out (ref)	-	-	-	-	-
	Internal or external hold-out	0.071	0.067	−0.081	0.223	0.318
Validation (group#2)	Whole data (ref)	-	-	-	-	-
	External	−0.074	0.161	−0.455	0.307	0.659
	Hold-out	−0.006	0.145	−0.349	0.337	0.967
	Cross-validation	−0.113	0.144	−0.453	0.226	0.457
WHO grade (group#1)	Grade 2, 3, or 4 (ref)	-	-	-	-	-
	Grade 4	−0.149	0.078	−0.328	0.031	0.092
	Grade 3 or 4	−0.067	0.088	−0.271	0.136	0.468
WHO grade (group#2)	Mixed (ref)	-	-	-	-	-
	High grade [grade 3 or 4] only	−0.121	0.074	−0.289	0.047	0.138
WHO grade (group#3)	Mixed (ref)	-	-	-	-	-
	Grade 4 only	−0.113	0.06	−0.249	0.024	0.095
METRICS (score)	-	8.562 × 10^−^^5^	0.003	−0.008	0.008	0.980
RQS (score)	-	2.401 × 10^−^^4^	0.003	−0.007	0.007	0.941
METRICS (category)	< Mean (ref)	-	-	-	-	-
	≥ Mean (high-quality)	−0.017	0.094	−0.231	0.196	0.861
RQS (category)	< Mean (ref)	-	-	-	-	-
	≥ Mean (high-quality)	0.017	0.071	−0.144	0.179	0.813

*WHO* World Health Organization, *METRICS* methodological radiomics score, *RQS* radiomics quality score, *ref* reference

**Fig. 6:**
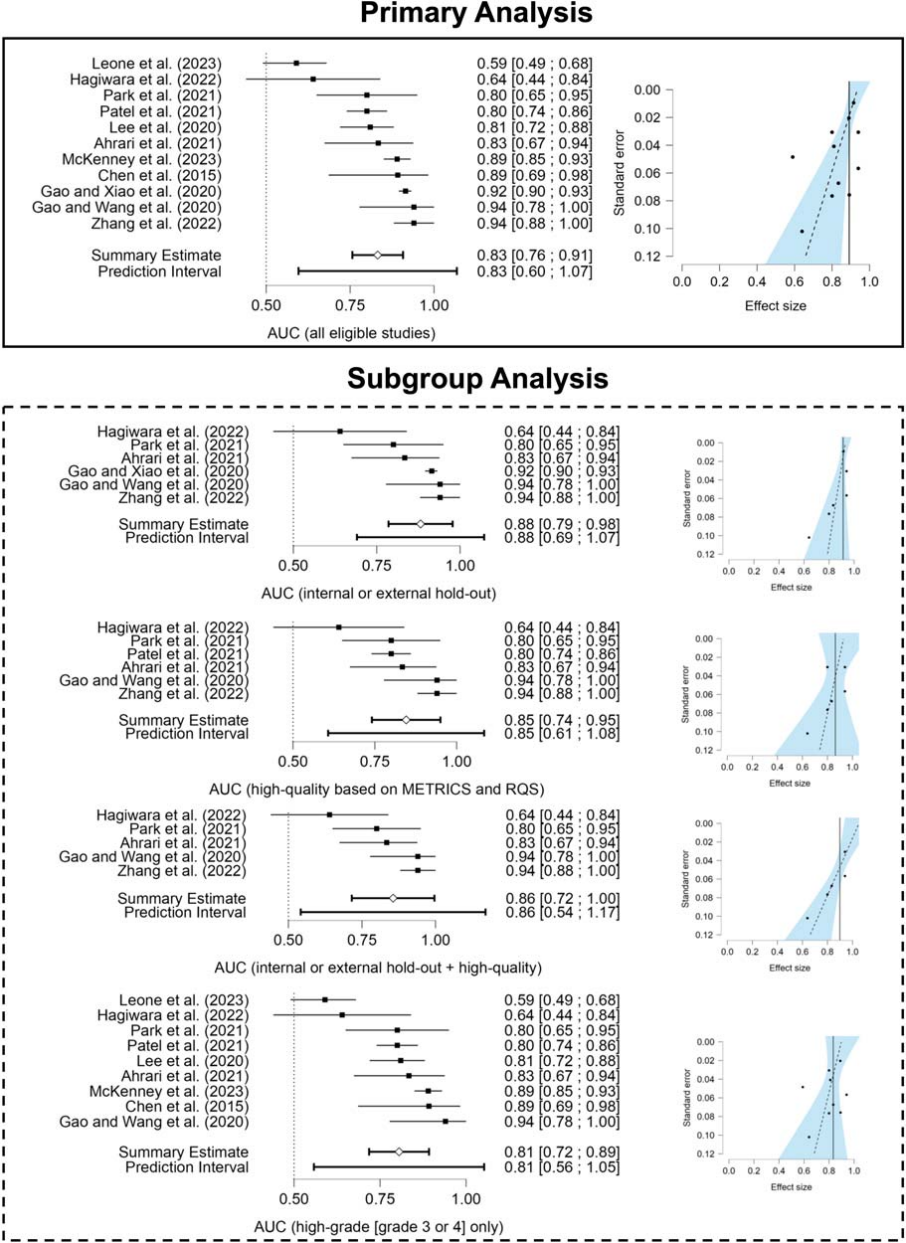
Funnel asymmetry and forest plots of the meta-analysis for all eligible studies (i.e., primary analysis) and study subgroups (i.e., subgroup analysis). AUC, area under the receiver operating characteristic curve; METRICS, methodological radiomics score; RQS, radiomics quality score

Further details of the meta-analysis results and funnel asymmetry plots are presented in Fig. 6 for both primary and subgroup analyses.

## Discussion

In this work, we systematically evaluated 27 glioma radiomics papers in differentiating radiation-induced brain injury from recurrence. We used two radiomics-specific quality scoring tools: very recent METRICS and well-established RQS [23, 24]. The methodological quality of papers was suboptimal, with a mean average METRICS score of 57% and a mean average RQS percentage score of 16%. Score-wise inter-rater agreement for METRICS ranged from poor to excellent, while RQS demonstrated moderate to excellent agreement. Item-wise agreement was moderate for both METRICS and RQS. The meta-analysis of 11 papers resulted in a favorable outcome, with an estimated AUC of 0.832. However, significant heterogeneity was observed and was not satisfactorily resolved in subsequent meta-regression or subgroup analyses. While statistical publication bias was generally not significant in primary and subgroup analyses, with *p*-values very close to the significance threshold in some of those, a subgroup analysis consisting of high-quality publications with separate internal or external hold-out validation data revealed statistical publication bias. Nevertheless, consistent with prior research on this topic [56], nearly all studies (26/27; 96%) reported positive effects of radiomics, indicating potential non-statistical publication bias.

In the literature, systematic reviews and meta-analyses on glioma radiomics for differentiating radiation-induced brain injury from recurrence are limited [57, 58]. Bhandari et al examined 25 studies on machine learning applications for distinguishing true tumor progression from treatment effects in brain tumors, including gliomas [57]. Their meta-analysis of nine glioma studies showed high sensitivity (95%) and specificity (82%) for distinguishing pseudoprogression from true tumor progression. However, these studies had a high risk of bias and moderate adherence to reporting guidelines (45%). The authors emphasized the need for better quality studies and integration into clinical practice, suggesting improvements in external validation and standardized reporting. Booth et al conducted a similar review on glioblastoma treatment response, analyzing 18 studies [58]. Most studies had a high risk of bias. Their meta-analysis of 10 studies yielded sensitivity, specificity, and AUC values of 0.769, 0.648, and 0.765, respectively. They concluded that while machine learning models performed well, study quality and design require significant improvement.

Regarding non-radiomics techniques, such as perfusion MRI, Zhang et al conducted a meta-analysis of 40 studies, including 1341 patients with glioma recurrence and 876 with pseudoprogression [15]. They reported pooled sensitivity and specificity values for DSC MR perfusion of 0.82 [95% confidence interval (CI): 0.78 to 0.86] and 0.87 [95% CI: 0.80 to 0.92], respectively, with an AUC of 0.89 [95% CI: 0.86 to 0.92]. Similarly, Wang et al performed a meta-analysis of 20 studies involving 939 patients and 968 lesions to assess post-treatment radiation effect and tumor progression in patients with glioma [59], reporting pooled sensitivity and specificity for DSC MR perfusion of 0.83 [95% CI: 0.79 to 0.86] and 0.83 [95% CI: 0.78 to 0.87], with an AUC of 0.887. Both meta-analyses concluded that DSC MR perfusion is a reliable option for assessing tumor progression following glioma radiotherapy. In our study, we observed comparable AUC values for radiomics approaches.

Our work has the following major strengths and related implications. First, we utilized two quality evaluation tools specifically designed for radiomics, which had not been previously applied to this particular topic. This is significant due to the complex nature of radiomics, requiring specialized assessment tools like METRICS and RQS, which offer complementary insights [25]. METRICS is a recent initiative, and this study is among its first use cases in radiomics [60–68]. Second, we provided a detailed item-wise analysis of these tools, identifying methodological deficiencies and suggesting specific improvements for future studies. These suggestions aim to address the challenges that may have so far hindered the clinical translation of radiomics. Examples include incorporating reporting guidelines at the study design stage (e.g., the CLEAR guideline [69]), assessing the robustness of deep learning studies (e.g., by evaluation of the consistency of performance in a test-retest setting, for example, by a scan-rescan approach, image perturbations, etc.), and effectively handling of confounding factors. Finally, we evaluated the item-wise reliability of these scoring tools, offering valuable feedback for their developers and aiding the creation of explanation and elaboration papers similar to Explanation and Elaboration with Examples for CLEAR (i.e., CLEAR-E3) [70] for CheckList for EvaluAtion of Radiomics research (i.e., CLEAR) [69].

We have a few limitations that might be useful to improve in future works. First, reporting quality was not evaluated for which CLEAR and CLAIM would be more suitable [69, 71]. Second, a specific bias assessment tool was not used as METRICS was already designed for this purpose and includes several items for evaluating bias sources relevant to radiomics. Third, the readers had different experience levels, which might impact the reliability metrics, and the quality assessments reported in this study. However, the reproducibility issues of such tools are already known [72]. To account for this, we presented different metrics to provide contextual interpretation. Nevertheless, the reliability of these tools should be analyzed and compared with optimal experimental settings as done in previous efforts [72]. Fourth, the methodological quality of the deep learning studies might be underestimated with RQS. Fifth, some baseline characteristics may be outdated or inappropriate due to the evolving nature of grading systems over time. For example, the WHO classification has expanded beyond traditional histological evaluation, incorporating molecular features into its grading criteria, which may not have been accounted for in older studies. Finally, we did not adopt a consensus approach when evaluating with quality scoring tools. Instead, we transparently presented all readers’ evaluation results as they are; and used averaging and collective (or cumulative) approaches to gain similar insights.

In conclusion, while the meta-analysis results show promise for radiomics, it is important to interpret them cautiously due to significant heterogeneity, statistical or non-statistical publication bias, and quality issues thoroughly examined in this study. Our findings highlight that, without a thorough bias (both statistical and non-statistical) and quality assessment, meta-analysis results can be misleading. In particular, negative results, especially those comparing radiomics with established reference methods, should be given more consideration in the publication landscape [56]. Incorporating the quality criteria outlined in METRICS and RQS tools during the design of future studies can lead to more robust and reliable outcomes, improving their potential for translation into clinical practice. Additionally, since self-reported quality scores tend to be higher than those assessed by external reviewers [73], the possibility of reporting bias should be addressed by utilizing radiomics-specific reporting guidelines such as CLEAR during the design phase of the studies. Finally, future versions of the quality scoring tools, along with their potential explanation and elaboration documents (e.g., CLEAR-E3 document [70] of CLEAR guideline [69]), might benefit from the extensive reliability analysis conducted as part of this work.

## Supplementary information

Electronic Supplementary Material

## Abbreviations

AUC: Area under the receiver operating characteristic curve
CI: Confidence interval
DSC: Dynamic susceptibility contrast
ICC: Intraclass correlation
METRICS: METhodological RadiomICs Score
RQS: Radiomics Quality Score
