# Supplementary material for: Radiomics for differentiating radiation-induced brain injury from recurrence in gliomas: systematic review, meta-analysis, and methodological quality evaluation using METRICS and RQS
Source: Eur Radiol. 2025 Feb 12;35(8):4490–505. doi: 10.1007/s00330-025-11401-x (PMC12226684; doi:10.1007/s00330-025-11401-x)
Supplement: Supplementary file 1 — Electronic Supplementary Material [file 330_2025_11401_MOESM1_ESM.pdf]

Electronic Supplementary Material

Radiomics for differentiating radiation-induced brain injury from recurrence in gliomas: systematic review, meta-analysis, and methodological quality evaluation using METRICS and RQS

Content

Supplementary Table S1. Additional baseline characteristics of 27 eligible papers.
Supplementary Figure S1. METRICS evaluation of reader 1.
Supplementary Figure S2. METRICS evaluation of reader 2.
Supplementary Figure S3. METRICS evaluation of reader 3.
Supplementary Figure S4. RQS evaluation of reader 1.
Supplementary Figure S5. RQS evaluation of reader 2.
Supplementary Figure S6. RQS evaluation of reader 3
Supplementary Checklist S1. PRISMA-DTA.
Supplementary Checklist S2. PRISMA-DTA for abstract.

Supplementary Table S1. Additional baseline characteristics of 27 eligible papers.

Authors (year)	WHO grade	Age range	Treatment for all patients	Detail on sequence or tracer
Ahrari et al. (2021)	3 and 4	Adult	RT but others unclear	18F-DOPA PET
Elshafeey et al. (2019)	4	Adult	RT + CTx (TMZ)	Perfusion MRI
Gao and Wang et al. (2020)	3 and 4	Adult	RT + CTx (TMZ)	Traditional MRI
Hagiwara et al. (2022)	4	Adult	RT + CTx (TMZ)	Traditional MRI
Kim et al. (2019)	4	Adult	RT + CTx (TMZ)	Traditional MRI + DWI + PWI
Lohmann et al. (2020)	3 and 4	Adult	RT + CTx (TMZ)	FET PET
Park et al. (2021)	4	Adult	RT + CTx (TMZ; not all)	Traditional MRI + DWI
Sun et al. (2021)	4	Adult + Pediatric (lower range 17)	RT + CTx (TMZ)	Traditional MRI
Wang et al. (2020)	2, 3, and 4	Adult	RT + CTx (TMZ)	Traditional MRI + FDG PET + MET PET
Zhang et al. (2019)	1, 2, 3, and 4	Adult + Pediatric (lower range 10)	RT but others unclear	Traditional MRI
Zhang et al. (2022)	2; 3; and 4	Adult	RT + CTx (TMZ)	Traditional MRI + DWI + PWI
Chen et al. (2015)	4	Unclear	RT + CTx (TMZ)	Traditional MRI
Akbari et al. (2020)	4	Adult	RT + CTx (TMZ)	Traditional MRI + DTI + PWI
Bacchi et al. (2019)	3 and 4	Adult	Unclear	Traditional MRI + DWI
Gao and Xiao et al. (2020)	2; 3; and 4	Adult	RT but others unclear	Traditional MRI

Jang et al. (2018)	4	Adult	RT + CTx (TMZ)	Traditional MRI
Kebir et al. (2017)	3 and 4	Adult	RT + CTx	FET PET
Lee et al. (2020)	3 and 4	Adult	RT + CTx	Traditional MRI + DWI
Li et al. (2020)	4	Unclear	RT + CTx (TMZ)	DTI
Moassefi et al. (2022)	4	Adult	RT + CTx (TMZ)	Traditional MRI
Patel et al. (2021)	4	Adult	RT + CTx (TMZ)	Traditional MRI + DWI
Siakallis et al. (2021)	3 and 4	Adult	RT + CTx	Traditional MRI + PWI
Zhang et al. (2016)	4	Unclear	RT + CTx	DTI
Leone et al. (2023)	4	Adult	RT + CTx (TMZ)	Traditional MRI + DWI
McKenney et al. (2023)	4	Adult	RT + CTx (TMZ)	Traditional MRI + PWI
Bani-Sadr et. al (2019)	4	Adult	RT + CTx (TMZ)	Traditional MRI
Jang et al. (2020)	4	Adult	RT + CTx	Traditional MRI

WHO, World Health Organization; **RT**, radiation therapy; **CTx**, chemotherapy, **TMZ**, temozolomide; **DWI**, diffusion-weighted imaging; **PWI**, perfusion-weighted imaging; **DTI**, diffusion tensor imaging

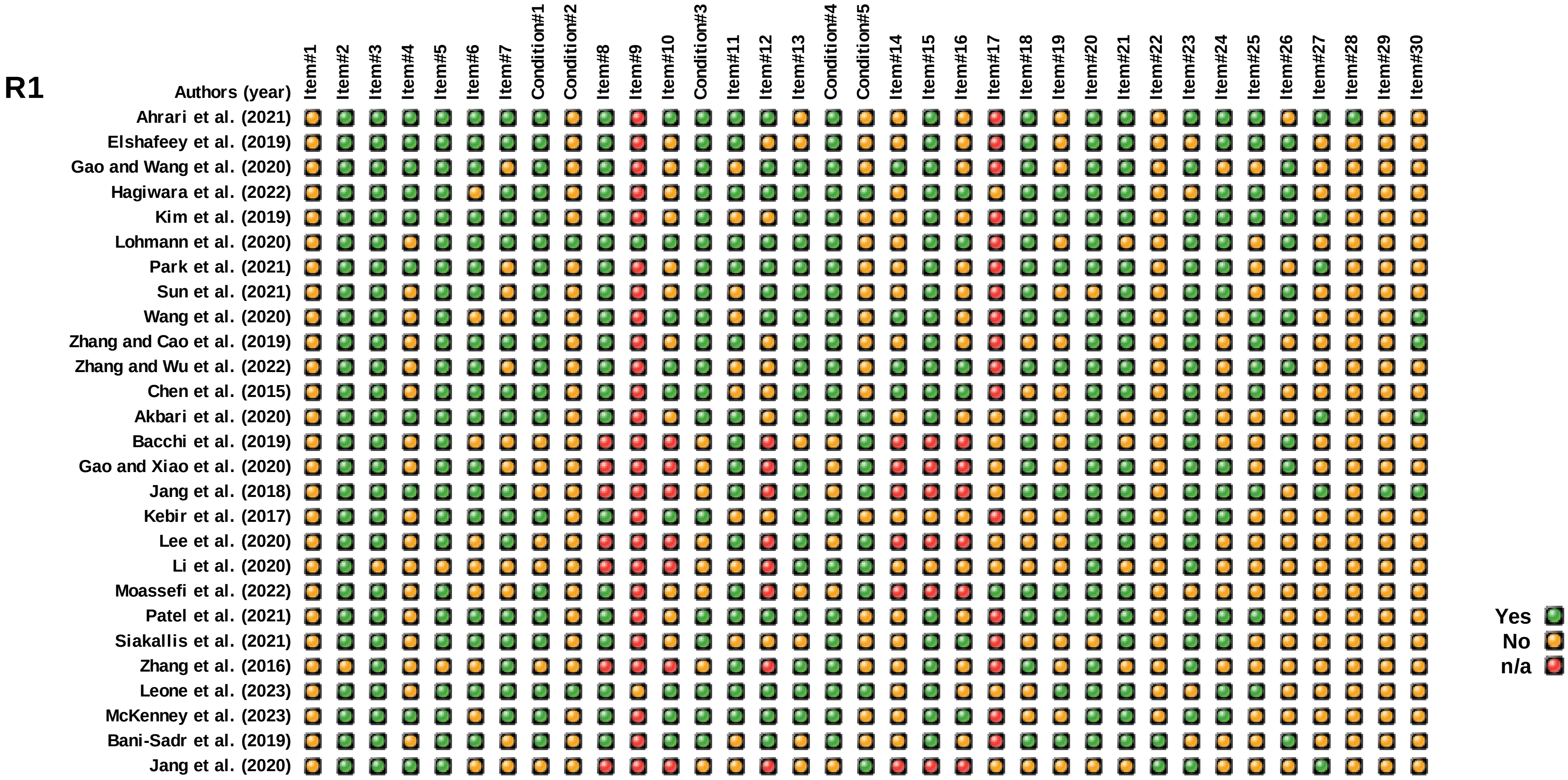

Supplementary Figure S1. METRICS evaluation of reader 1.

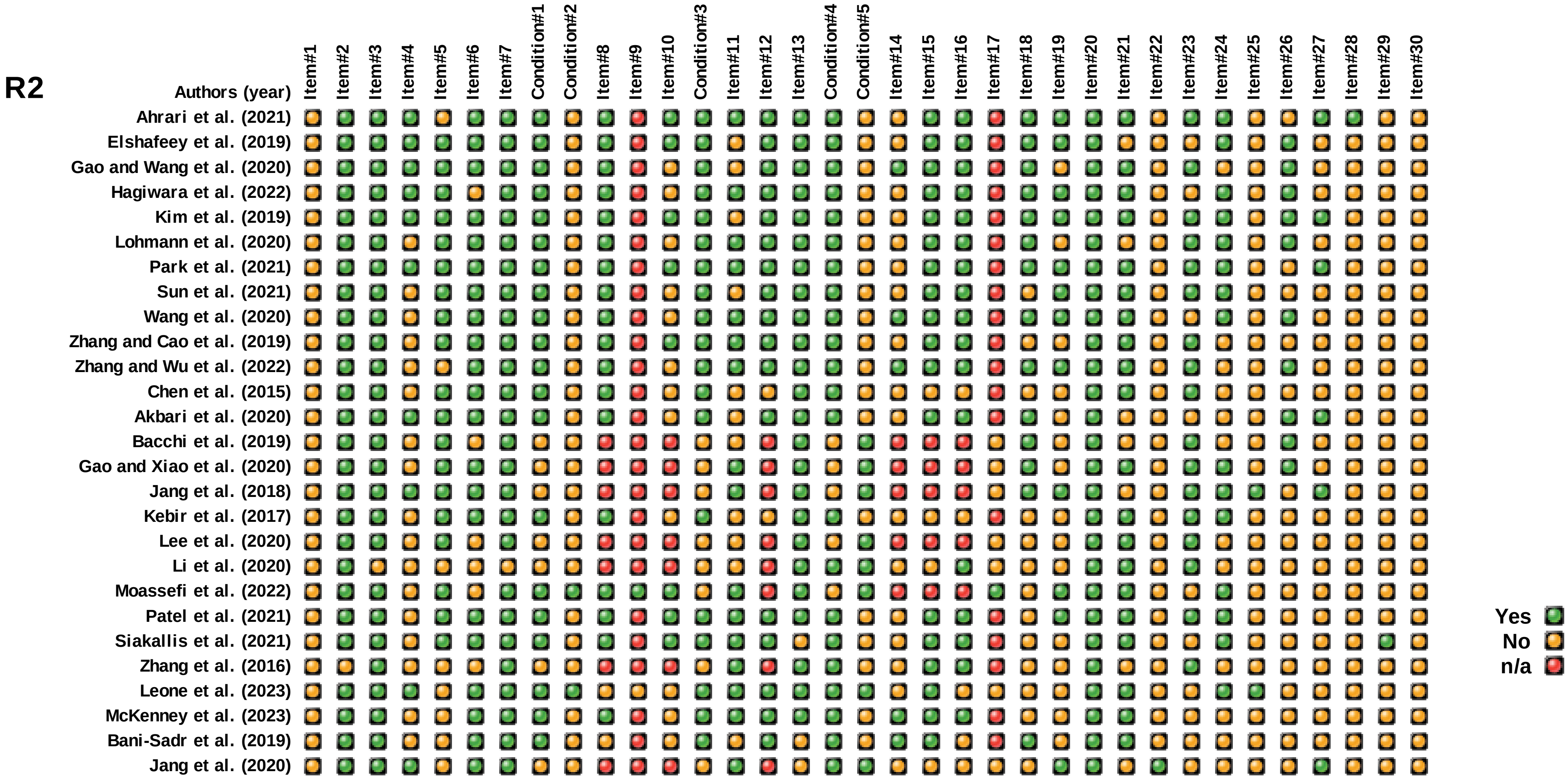

Supplementary Figure S2. METRICS evaluation of reader 2.

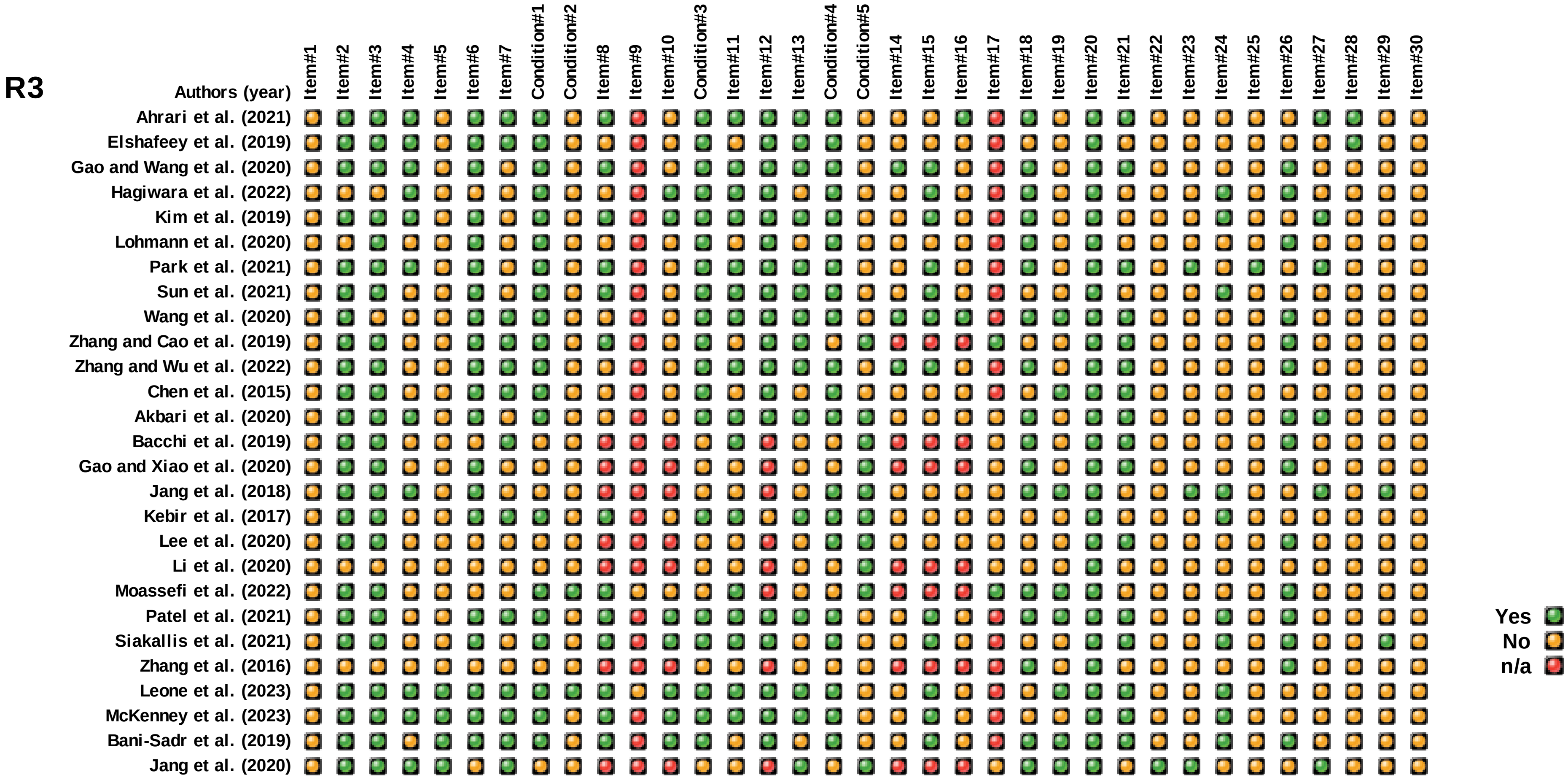

Supplementary Figure S3. METRICS evaluation of reader 3.

R1

Authors (year)	Image protocol quality	Multiple segmentations	Phantom study on all scanners	Imaging at multiple time points	Feature reduction or adjustment for multiple testing	Multivariable analysis with non-radiomics features	Detect and discuss biological correlates	Cut-off analyses	Discrimination statistics	Calibration statistics	Prospective study registered in a trial database	Validation	Comparison to 'gold standard'	Potential clinical utility	Cost-effectiveness analysis	Open science and data
Ahrari et al. (2021)	1	0	0	0	3	1	0	1	2	0	0	3	2	0	0	0
Elshafeey et al. (2019)	1	0	0	0	3	0	0	0	2	0	0	2	2	0	0	0
Gao and Wang et al. (2020)	1	1	0	0	3	0	0	0	2	0	0	2	0	0	0	0
Hagiwara et al. (2022)	0	0	0	0	3	1	0	1	2	0	0	2	2	0	0	0
Kim et al. (2019)	1	0	0	0	3	1	0	0	2	0	0	4	2	0	0	0
Lohmann et al. (2020)	1	1	0	0	3	0	0	0	2	0	0	2	2	0	0	0
Park et al. (2021)	1	0	0	0	3	0	0	0	2	0	0	3	2	0	0	0
Sun et al. (2021)	1	0	0	0	3	0	0	0	0	0	0	2	2	0	0	0
Wang et al. (2020)	0	1	0	0	3	1	0	1	2	0	0	2	0	2	0	0
Zhang and Cao et al. (2019)	1	0	0	0	3	0	0	0	2	0	0	-5	0	0	0	0
Zhang and Wu et al. (2022)	1	1	0	0	3	0	0	0	2	0	0	2	0	0	0	0
Chen et al. (2015)	1	1	0	0	-3	0	0	1	1	0	0	-5	0	0	0	0
Akbari et al. (2020)	1	0	0	0	3	0	1	0	2	0	0	3	0	0	0	0
Bacchi et al. (2019)	0	0	0	0	-3	0	0	0	2	0	0	2	0	0	0	0
Gao and Xiao et al. (2020)	1	0	0	0	-3	0	0	0	1	0	0	2	2	0	0	0
Jang et al. (2018)	1	0	0	0	-3	1	0	0	2	0	0	3	2	0	0	0
Kebir et al. (2017)	1	0	0	0	-3	1	1	1	0	0	0	-5	2	0	0	0
Lee et al. (2020)	0	0	0	0	-3	0	0	0	2	0	0	-5	0	0	0	0
Li et al. (2020)	0	0	0	0	-3	0	0	0	2	0	0	-5	0	0	0	0
Moassefi et al. (2022)	0	0	0	0	3	1	0	0	2	0	0	-5	0	0	0	0
Patel et al. (2021)	1	0	0	0	3	1	0	0	2	0	0	-5	2	0	0	0
Siakallis et al. (2021)	1	0	0	0	3	0	0	0	0	0	0	-5	2	0	0	0
Zhang et al. (2016)	0	0	0	0	3	0	0	0	2	0	0	-5	0	0	0	0
Leone et al. (2023)	1	0	0	0	3	1	0	0	2	0	0	-5	2	0	0	0
McKenney et al. (2023)	1	0	0	0	3	1	0	0	2	0	0	-5	2	0	0	0
Bani-Sadr et al. (2019)	1	0	0	0	3	1	0	1	1	1	0	2	0	0	0	0
Jang et al. (2020)	0	0	0	0	-3	1	0	0	0	2	0	4	0	0	0	0

Supplementary Figure S4. RQS evaluation of reader 1.

R2

Authors (year)	Image protocol quality	Multiple segmentations	Phantom study on all scanners	Imaging at multiple time points	Feature reduction or adjustment for multiple testing	Multivariable analysis with non-radiomics features	Detect and discuss biological correlates	Cut-off analyses	Discrimination statistics	Calibration statistics	Prospective study registered in a trial database	Validation	Comparison to ‘gold standard’	Potential clinical utility	Cost-effectiveness analysis	Open science and data
Ahrari et al. (2021)	1	0	1	0	3	1	0	0	2	0	0	3	2	0	0	0
Elshafeey et al. (2019)	1	0	0	0	3	1	0	0	2	0	0	2	2	0	0	0
Gao and Wang et al. (2020)	1	1	0	0	3	0	0	0	2	0	0	2	0	0	0	0
Hagiwara et al. (2022)	0	0	0	0	3	1	0	0	2	0	0	2	0	0	0	0
Kim et al. (2019)	1	0	0	0	3	1	0	0	2	0	0	4	2	0	0	0
Lohmann et al. (2020)	1	0	0	0	3	1	0	0	2	0	0	2	2	0	0	0
Park et al. (2021)	1	0	0	0	3	0	0	0	2	0	0	3	0	0	0	0
Sun et al. (2021)	1	1	0	0	3	0	0	0	2	0	0	-5	2	0	0	0
Wang et al. (2020)	1	1	0	0	3	1	0	1	2	0	0	2	2	2	0	0
Zhang and Cao et al. (2019)	1	0	0	0	3	0	0	0	2	0	0	-5	0	0	0	0
Zhang and Wu et al. (2022)	1	1	0	0	3	0	0	0	2	0	0	2	0	0	0	0
Chen et al. (2015)	1	0	0	0	-3	0	0	1	0	0	0	-5	0	0	0	0
Akbari et al. (2020)	1	0	0	0	3	0	1	0	2	0	0	4	2	0	0	0
Bacchi et al. (2019)	0	0	0	0	-3	0	0	0	2	0	0	2	0	0	0	0
Gao and Xiao et al. (2020)	1	0	0	0	-3	0	0	0	1	0	0	2	2	0	0	0
Jang et al. (2018)	1	0	0	0	-3	1	0	0	2	0	0	3	0	0	0	0
Kebir et al. (2017)	1	0	0	0	-3	1	0	1	1	0	0	-5	2	0	0	0
Lee et al. (2020)	0	0	0	0	-3	0	0	0	2	0	0	-5	0	0	0	0
Li et al. (2020)	0	0	0	0	-3	0	0	0	2	0	0	-5	0	0	0	0
Moassefi et al. (2022)	0	0	0	0	-3	1	0	0	2	0	0	-5	0	0	0	0
Patel et al. (2021)	1	0	0	0	3	1	0	0	2	0	0	-5	2	0	0	0
Siakallis et al. (2021)	1	0	0	1	3	1	0	0	2	0	0	-5	2	0	0	1
Zhang et al. (2016)	0	0	0	0	3	0	0	0	2	0	0	-5	0	0	0	0
Leone et al. (2023)	1	0	0	0	3	1	1	1	2	0	0	-5	2	0	0	0
McKenney et al. (2023)	1	0	0	0	3	1	0	0	2	0	0	-5	0	0	0	0
Bani-Sadr et al. (2019)	1	0	0	0	3	1	0	0	2	0	0	2	0	0	0	0
Jang et al. (2020)	0	0	0	0	-3	1	0	0	2	1	0	3	0	0	0	0

Supplementary Figure S5. RQS evaluation of reader 2.

R3

Authors (year)	Image protocol quality	Multiple segmentations	Phantom study on all scanners	Imaging at multiple time points	Feature reduction or adjustment for multiple testing	Multivariable analysis with non-radiomics features	Detect and discuss biological correlates	Cut-off analyses	Discrimination statistics	Calibration statistics	Prospective study registered in a trial database	Validation	Comparison to 'gold standard'	Potential clinical utility	Cost-effectiveness analysis	Open science and data
Ahrari et al. (2021)	1	0	0	0	3	0	0	0	2	1	0	3	2	0	0	1
Elshafeey et al. (2019)	1	0	0	0	-3	0	0	0	1	0	0	2	0	0	0	1
Gao and Wang et al. (2020)	1	1	0	0	3	0	0	0	2	1	0	2	0	0	0	0
Hagiwara et al. (2022)	0	0	0	0	3	1	0	0	1	0	0	2	0	0	0	0
Kim et al. (2019)	1	0	0	0	3	1	0	0	1	0	0	3	2	0	0	0
Lohmann et al. (2020)	1	0	0	0	3	0	0	1	2	1	0	2	2	0	0	0
Park et al. (2021)	1	0	0	0	3	0	0	0	2	1	0	3	0	0	0	0
Sun et al. (2021)	1	0	0	0	3	1	0	0	2	1	0	-5	2	0	0	0
Wang et al. (2020)	1	1	0	0	3	0	0	0	2	1	0	2	0	0	0	0
Zhang and Cao et al. (2019)	1	0	0	0	3	0	0	0	2	1	0	2	0	0	0	0
Zhang and Wu et al. (2022)	1	1	0	0	3	0	0	0	2	1	0	2	0	0	0	0
Chen et al. (2015)	1	0	0	0	-3	0	0	1	1	0	0	-5	0	0	0	0
Akbari et al. (2020)	1	0	0	0	-3	0	0	0	1	0	0	3	0	0	0	0
Bacchi et al. (2019)	0	0	0	0	-3	0	0	0	1	0	0	2	0	0	0	0
Gao and Xiao et al. (2020)	1	0	0	0	-3	0	0	0	1	0	0	2	0	0	0	0
Jang et al. (2018)	1	0	0	0	-3	1	0	0	1	0	0	3	0	0	0	1
Kebir et al. (2017)	1	0	0	0	-3	0	0	0	1	0	0	-5	0	0	0	0
Lee et al. (2020)	0	0	0	0	-3	0	0	0	1	0	0	2	0	0	0	0
Li et al. (2020)	0	0	0	0	-3	0	0	0	1	0	0	-5	0	0	0	0
Moassefi et al. (2022)	0	0	0	0	3	0	0	0	2	1	0	2	0	0	0	0
Patel et al. (2021)	1	0	0	0	3	1	0	0	2	1	0	2	0	0	0	0
Siakallis et al. (2021)	1	0	0	0	3	1	0	0	2	1	0	2	2	0	0	1
Zhang et al. (2016)	0	0	0	0	-3	0	0	0	2	1	0	2	0	0	0	0
Leone et al. (2023)	1	0	0	0	3	1	0	0	2	0	0	-5	0	0	0	0
McKenney et al. (2023)	1	0	0	0	3	1	0	0	2	0	0	-5	2	0	0	0
Bani-Sadr et al. (2019)	1	0	0	0	3	1	0	0	2	0	0	2	0	0	0	0
Jang et al. (2020)	1	0	0	0	-3	1	0	0	2	1	0	4	0	0	0	0

Supplementary Figure S6. RQS evaluation of reader 3.

Supplementary Checklist S1. PRISMA-DTA.

Section/topic	#	PRISMA-DTA Checklist Item	Reported on page #
**TITLE / ABSTRACT**			
Title	1	Identify the report as a systematic review (+/- meta-analysis) of diagnostic test accuracy (DTA) studies.	Title
Abstract	2	Abstract: See PRISMA-DTA for abstracts.	Seen
**INTRODUCTION**			
Rationale	3	Describe the rationale for the review in the context of what is already known.	Intro
Clinical role of index test	D1	State the scientific and clinical background, including the intended use and clinical role of the index test, and if applicable, the rationale for minimally acceptable test accuracy (or minimum difference in accuracy for comparative design).	Intro
Objectives	4	Provide an explicit statement of question(s) being addressed in terms of participants, index test(s), and target condition(s).	Intro
**METHODS**			
Protocol and registration	5	Indicate if a review protocol exists, if and where it can be accessed (e.g., Web address), and, if available, provide registration information including registration number.	None
Eligibility criteria	6	Specify study characteristics (participants, setting, index test(s), reference standard(s), target condition(s), and study design) and report characteristics (e.g., years considered, language, publication status) used as criteria for eligibility, giving rationale.	Methods
Information sources	7	Describe all information sources (e.g., databases with dates of coverage, contact with study authors to identify additional studies) in the search and date last searched.	Methods
Search	8	Present full search strategies for all electronic databases and other sources searched, including any limits used, such that they could be repeated.	Methods + Figure 1
Study selection	9	State the process for selecting studies (i.e., screening, eligibility, included in systematic review, and, if applicable, included in the meta-analysis).	Methods
Data collection process	10	Describe method of data extraction from reports (e.g., piloted forms, independently, in duplicate) and any processes for obtaining and confirming data from investigators.	Methods
Definitions for data extraction	11	Provide definitions used in data extraction and classifications of target condition(s), index test(s), reference standard(s) and other characteristics (e.g. study design, clinical setting).	Methods
Risk of bias and applicability	12	Describe methods used for assessing risk of bias in individual studies and concerns regarding the applicability to the review question.	Methods (with quality scoring

			tools)
Diagnostic accuracy measures	13	State the principal diagnostic accuracy measure(s) reported (e.g. sensitivity, specificity) and state the unit of assessment (e.g. per-patient, per-lesion).	Methods
Synthesis of results	14	Describe methods of handling data, combining results of studies and describing variability between studies. This could include, but is not limited to: a) handling of multiple definitions of target condition. b) handling of multiple thresholds of test positivity, c) handling multiple index test readers, d) handling of indeterminate test results, e) grouping and comparing tests, f) handling of different reference standards	Methods

Section/topic	#	PRISMA-DTA Checklist Item	Reported on page #
Meta-analysis	D2	Report the statistical methods used for meta-analyses, if performed.	Methods
Additional analyses	16	Describe methods of additional analyses (e.g., sensitivity or subgroup analyses, meta-regression), if done, indicating which were pre-specified.	Methods
**RESULTS**			
Study selection	17	Provide numbers of studies screened, assessed for eligibility, included in the review (and included in meta-analysis, if applicable) with reasons for exclusions at each stage, ideally with a flow diagram.	Results
Study characteristics	18	For each included study provide citations and present key characteristics including: a) participant characteristics (presentation, prior testing), b) clinical setting, c) study design, d) target condition definition, e) index test, f) reference standard, g) sample size, h) funding sources	Results, Table 1
Risk of bias and applicability	19	Present evaluation of risk of bias and concerns regarding applicability for each study.	Results, Fig 2-3-4-5
Results of individual studies	20	For each analysis in each study (e.g. unique combination of index test, reference standard, and positivity threshold) report 2x2 data (TP, FP, FN, TN) with estimates of diagnostic accuracy and confidence intervals, ideally with a forest or receiver operator characteristic (ROC) plot.	Fig 6
Synthesis of results	21	Describe test accuracy, including variability; if meta-analysis was done, include results and confidence intervals.	Fig 6
Additional analysis	23	Give results of additional analyses, if done (e.g., sensitivity or subgroup analyses, meta-regression; analysis of index test: failure rates, proportion of inconclusive results, adverse events).	Results Table 5
**DISCUSSION**			
Summary of evidence	24	Summarize the main findings including the strength of evidence.	Disc

Limitations	25	Discuss limitations from included studies (e.g. risk of bias and concerns regarding applicability) and from the review process (e.g. incomplete retrieval of identified research).	Disc
Conclusions	26	Provide a general interpretation of the results in the context of other evidence. Discuss implications for future research and clinical practice (e.g. the intended use and clinical role of the index test).	Disc
**FUNDING**			
Funding	27	For the systematic review, describe the sources of funding and other support and the role of the funders.	N/a

Adapted From: McInnes MDF, Moher D, Thombs BD, McGrath TA, Bossuyt PM, The PRISMA-DTA Group (2018). Preferred Reporting Items for a Systematic Review and Meta-analysis of Diagnostic Test Accuracy Studies: The PRISMA-DTA Statement. JAMA. 2018 Jan 23;319(4):388-396. doi: 10.1001/jama.2017.19163.

For more information, visit: **www.prisma-statement.org**.

Supplementary Checklist S2. PRISMA-DTA for abstract.

Section/topic	#	PRISMA-DTA for Abstracts Checklist item	Reported on page #
**TITLE and PURPOSE**			
Title	1	Identify the report as a systematic review (+/- meta-analysis) of diagnostic test accuracy (DTA) studies.	Title
Objectives	2	Indicate the research question, including components such as participants, index test, and target conditions.	Objective
**METHODS**			
Eligibility criteria	3	Include study characteristics used as criteria for eligibility.	Objective
Information sources	4	List the key databases searched and the search dates.	Methods
Risk of bias & applicability	5	Indicate the methods of assessing risk of bias and applicability.	Methods
Synthesis of results	A1	Indicate the methods for the data synthesis.	Methods
**RESULTS**			
Included studies	6	Indicate the number and type of included studies and the participants and relevant characteristics of the studies (including the reference standard).	Results
Synthesis of results	7	Include the results for the analysis of diagnostic accuracy, preferably indicating the number of studies and participants. Describe test accuracy including variability; if meta-analysis was done, include summary results and confidence intervals.	Results
**DISCUSSION**			
Strengths and limitations	9	Provide a brief summary of the strengths and limitations of the evidence	Conclusion, Clinical Relevance
Interpretation	10	Provide a general interpretation of the results and the important implications.	Conclusion, Clinical Relevance
**OTHER**			
Funding	11	Indicate the primary source of funding for the review.	N/a
Registration	12	Provide the registration number and the registry name	N/a

Adapted From: McInnes MDF, Moher D, Thombs BD, McGrath TA, Bossuyt PM, The PRISMA-DTA Group (2018). Preferred Reporting Items for a Systematic Review and Meta-analysis of Diagnostic Test Accuracy Studies: The PRISMA-DTA Statement. JAMA. 2018 Jan 23;319(4):388-396. doi: 10.1001/jama.2017.19163.
